# Supplementary material for: Effects of perioperative massive transfusion on postoperative outcomes of children undergoing brain tumor removal: a retrospective cohort study
Source: PeerJ. 2025 May 13;13:e19350. doi: 10.7717/peerj.19350 (PMC12083467; doi:10.7717/peerj.19350)
Supplement: Supplemental Information 2 [file peerj-13-19350-s002.docx]

Diagnosis of postoperative new neurologic events is based on the International Statistical Classification of Diseases and Related Health Problems, 10th Revision (ICD-10).1 The following are examples of criteria of neurological events diagnosed in the patients involved this study.

References:

1. World Health Organization. (2017). International Classification of Diseases. [online]. Available from <http://www.who.int/classifications/icd/en/>

**Table S1 Definitions of postoperative new neurologic events**

| **Category** | **Diagnosis** |
| --- | --- |
| Focal seizure | EEG evidence + clinical symptoms and signs |
| Muscle weakness | Muscle grade assessment for four limbs according to the Medical Research Council scale2 |
| Disorientation | A decrease of Glasgow Coma Scale (GCS) comparing to preoperative status  A mental state marked by confusion about time (temporal), place (spatial), or who one is.  Agitation, hallucination (visual or hearing) |
| Aphasia | A cognitive disorder marked by an impaired ability to comprehend or express language in its written or spoken form. |
| Visual disturbance | Anopsia, amblyopia…etc. |
