# Supplementary material for: Effects of perioperative massive transfusion on postoperative outcomes of children undergoing brain tumor removal: a retrospective cohort study
Source: PeerJ. 2025 May 13;13:e19350. doi: 10.7717/peerj.19350 (PMC12083467; doi:10.7717/peerj.19350)
Supplement: Supplemental Information 3 [file peerj-13-19350-s003.docx]

**Table S2 Multivariable cox regression analysis of survival.**

| Variables | ***p* Value** | HR | 95% CI |
| --- | --- | --- | --- |
| WHO grade | 0.000 | 1.817 | 1.568 ~ 2.104 |
| Preoperative KPS score | 0.986 | 1.000 | 0.981 ~ 1.019 |
| Massive transfusion | 0.010 | 1.001 | 1.000 ~ 1.003 |
| Age (month) | 0.612 | 0.987 | 0.938 ~ 1.039 |
| Brain tumor resection score | 0.491 | 0.176 | 0.001 ~ 24.854 |

KPS, Karnofsky performance scale; WHO, World Health Organization. *p<0.05 **p<0.01.
