# Supplementary material for: Effects of perioperative massive transfusion on postoperative outcomes of children undergoing brain tumor removal: a retrospective cohort study
Source: PeerJ. 2025 May 13;13:e19350. doi: 10.7717/peerj.19350 (PMC12083467; doi:10.7717/peerj.19350)
Supplement: Supplemental Information 4 [file peerj-13-19350-s004.docx]

| **Table S3 Multivariable logistic regression analysis of postoperative secondary outcomes** | | | | |
| --- | --- | --- | --- | --- |
|  | **Variables** | **OR** | **95% CI** | ***p* Value** |
| **Infection** | |  |  |  |
|  | **Preoperative** |  |  |  |
|  | Massive transfusion | 0.693 | 0.273 ~ 1.761 | 0.441 |
|  | Male vs. female | 0.766 | 0.426 ~ 1.378 | 0.373 |
|  | Age (months) | 1.024 | 0.939 ~ 1.117 | 0.594 |
|  | Body weight (kg) | 0.910 | 0.842 ~ 0.984 | 0.017***** |
|  | Hydrocephalus surgical treatment | 0.915 | 0.424 ~ 1.977 | 0.821 |
|  | KPS score | 1.014 | 0.983 ~ 1.046 | 0.381 |
|  | Anaemia | 0.398 | 0.169 ~ 0.937 | 0.035***** |
|  | Coagulopathy | 0.637 | 0.189 ~ 2.145 | 0.467 |
|  | Edema | 0.630 | 0.333 ~ 1.189 | 0.154 |
|  | Subtentorial tumor | 1.600 | 0.845 ~ 3.027 | 0.149 |
|  | Tumor size(cm) | 1.034 | 0.874 ~ 1.224 | 0.697 |
|  | Tumor number (multiple) | 0.224 | 0.070 ~ 0.718 | 0.012* |
|  | Tumor invasiveness | 0.756 | 0.342 ~ 1.672 | 0.489 |
|  | Intraoperative |  |  |  |
|  | Brain tumor resection score | 0.713 | 0.488 ~ 1.044 | 0.082 |
|  | Crystalloids infusion (ml/kg) | 0.993 | 0.987 ~ 0.998 | 0.009****** |
|  | Colloids infusion (ml/kg) | 0.992 | 0.980 ~ 1.005 | 0.220 |
|  | Blood loss (ml/kg) | 1.006 | 1.001 ~ 1.011 | 0.019***** |
|  | Surgical time (min) | 1.003 | 1.000 ~ 1.007 | 0.086 |
|  | Postoperative |  |  |  |
|  | Ki67-proliferation rate | 1.013 | 0.999 ~ 1.028 | 0.077 |
|  | WHO grade | 1.007 | 0.741 ~ 1.369 | 0.963 |
| **Re-craniotomy** | |  |  |  |
|  | Preoperative |  |  |  |
|  | Massive transfusion | 2.617 | 0.050 ~ 136.317 | 0.633 |
|  | Male vs. female | 14.223 | 0.380 ~ 532.219 | 0.151 |
|  | Age (months) | 1.181 | 0.708 ~ 1.971 | 0.523 |
|  | Body weight (kg) | 0.972 | 0.634 ~ 1.490 | 0.895 |
|  | Hydrocephalus surgical treatment | 0.525 | 0.025 ~ 10.863 | 0.677 |
|  | KPS score | 0.902 | 0.762 ~ 1.069 | 0.234 |
|  | Anaemia | 0.295 | 0.002 ~ 48.508 | 0.639 |
|  | Coagulopathy | 0.000 | 0.000 ~ null | 0.999 |
|  | Edema | 12.071 | 0.313 ~ 465.840 | 0.181 |
|  | Subtentorial tumor | 0.022 | 0.000 ~ 2.057 | 0.099 |
|  | Tumor size(cm) | 0.682 | 0.283 ~ 1.643 | 0.394 |
|  | Tumor number (multiple) | 7.251 | 0.228 ~ 230.622 | 0.262 |
|  | Tumor invasiveness | 1.814 | 0.126 ~ 26.204 | 0.662 |
|  | Intraoperative |  |  |  |
|  | Brain tumor resection score | 1.339 | 0.276 ~ 6.497 | 0.717 |
|  | Crystalloids infusion (ml/kg) | 0.931 | 0.852 ~ 1.017 | 0.112 |
|  | Colloids infusion (ml/kg) | 1.060 | 1.003 ~ 1.119 | 0.038***** |
|  | Blood loss (ml/kg) | 1.017 | 0.996 ~ 1.039 | 0.121 |
|  | Surgical time (min) | 1.007 | 0.987 ~ 1.029 | 0.492 |
|  | Postoperative |  |  |  |
|  | Ki67-proliferation rate | 0.974 | 0.918 ~ 1.033 | 0.381 |
|  | WHO grade | 2.299 | 0.499 ~ 10.585 | 0.285 |
| **Intracranial hypertension** | |  |  |  |
|  | Preoperative |  |  |  |
|  | Massive transfusion | 4.788 | 1.547 ~ 14.824 | 0.007****** |
|  | Male vs. female | 1.754 | 0.818 ~ 3.759 | 0.149 |
|  | Age (months) | 1.082 | 0.961 ~ 1.218 | 0.195 |
|  | Body weight (kg) | 1.080 | 0.981 ~ 1.189 | 0.119 |
|  | Hydrocephalus surgical treatment | 1.130 | 0.434 ~ 2.941 | 0.802 |
|  | KPS score | 0.969 | 0.932 ~ 1.008 | 0.120 |
|  | Anaemia | 4.418 | 1.795 ~ 10.874 | 0.001****** |
|  | Coagulopathy | 0.229 | 0.022 ~ 2.399 | 0.219 |
|  | Edema | 1.397 | 0.596 ~ 3.275 | 0.441 |
|  | Subtentorial tumor | 0.635 | 0.272 ~ 1.479 | 0.293 |
|  | Tumor size(cm) | 1.228 | 0.981 ~ 1.537 | 0.074 |
|  | Tumor number (multiple) | 3.601 | 1.393 ~ 9.310 | 0.008****** |
|  | Tumor invasiveness | 2.532 | 1.070 ~ 5.992 | 0.035***** |
|  | Intraoperative |  |  |  |
|  | Brain tumor resection score | 1.353 | 0.918 ~ 1.992 | 0.126 |
|  | Crystalloids infusion (ml/kg) | 1.000 | 0.994 ~ 1.007 | 0.962 |
|  | Colloids infusion (ml/kg) | 1.006 | 0.993 ~ 1.019 | 0.391 |
|  | Blood loss (ml/kg) | 1.000 | 0.994 ~ 1.006 | 0.904 |
|  | Surgical time (min) | 0.995 | 0.990 ~ 1.000 | 0.041***** |
|  | Postoperative |  |  |  |
|  | Ki67-proliferation rate | 1.000 | 0.982 ~ 1.019 | 0.969 |
|  | WHO grade | 1.317 | 0.890 ~ 1.949 | 0.168 |
| **New neurologic events** | |  |  |  |
|  | Preoperative |  |  |  |
|  | Massive transfusion | 5.312 | 0.760 ~ 37.124 | 0.092 |
|  | Male vs. female | 0.659 | 0.180 ~ 2.408 | 0.528 |
|  | Age (months) | 0.911 | 0.745 ~ 1.114 | 0.365 |
|  | Body weight (kg) | 0.839 | 0.694 ~ 1.014 | 0.069 |
|  | Hydrocephalus surgical treatment | 3.958 | 0.918 ~ 17.064 | 0.065 |
|  | KPS score | 0.991 | 0.944 ~ 1.040 | 0.715 |
|  | Anaemia | 0.500 | 0.078 ~ 3.220 | 0.466 |
|  | Coagulopathy | 1.405 | 0.139 ~ 14.239 | 0.773 |
|  | Edema | 1.167 | 0.295 ~ 4.621 | 0.826 |
|  | Subtentorial tumor | 0.613 | 0.137 ~ 2.742 | 0.522 |
|  | Tumor size(cm) | 0.606 | 0.372 ~ 0.987 | 0.044***** |
|  | Tumor number (multiple) | 2.819 | 0.656 ~ 12.106 | 0.163 |
|  | Tumor invasiveness | 6.191 | 1.505 ~ 25.475 | 0.012***** |
|  | Intraoperative |  |  |  |
|  | Brain tumor resection score | 0.534 | 0.214 ~ 1.332 | 0.179 |
|  | Crystalloids infusion (ml/kg) | 0.988 | 0.965 ~ 1.012 | 0.323 |
|  | Colloids infusion (ml/kg) | 0.996 | 0.966 ~ 1.028 | 0.820 |
|  | Blood loss (ml/kg) | 0.992 | 0.973 ~ 1.012 | 0.427 |
|  | Surgical time (min) | 1.000 | 0.990 ~ 1.010 | 0.954 |
|  | Postoperative |  |  |  |
|  | Ki67-proliferation rate | 1.002 | 0.970 ~ 1.035 | 0.920 |
|  | WHO grade | 0.945 | 0.475 ~ 1.881 | 0.872 |
| **Recurrence or metastasis** | |  |  |  |
|  | Preoperative |  |  |  |
|  | Massive transfusion | 0.103 | 0.007 ~ 1.422 | 0.090 |
|  | Male vs. female | 0.901 | 0.292 ~ 2.787 | 0.857 |
|  | Age (months) | 0.987 | 0.831 ~ 1.172 | 0.882 |
|  | Body weight (kg) | 0.987 | 0.870 ~ 1.119 | 0.835 |
|  | Hydrocephalus surgical treatment | 0.633 | 0.116 ~ 3.465 | 0.598 |
|  | KPS score | 0.957 | 0.908 ~ 1.009 | 0.106 |
|  | Anaemia | 1.635 | 0.427 ~ 6.257 | 0.472 |
|  | Coagulopathy | 1.066 | 0.109 ~ 10.423 | 0.956 |
|  | Edema | 0.399 | 0.118 ~ 1.346 | 0.139 |
|  | Subtentorial tumor | 0.754 | 0.220 ~ 2.581 | 0.653 |
|  | Tumor size(cm) | 1.182 | 0.875 ~ 1.597 | 0.276 |
|  | Tumor number (multiple) | 2.337 | 0.524 ~ 10.426 | 0.266 |
|  | Tumor invasiveness | 0.887 | 0.227 ~ 3.470 | 0.864 |
|  | Intraoperative |  |  |  |
|  | Brain tumor resection score | 0.586 | 0.234 ~ 1.469 | 0.254 |
|  | Crystalloids infusion (ml/kg) | 0.983 | 0.965 ~ 1.001 | 0.060 |
|  | Colloids infusion (ml/kg) | 1.008 | 0.985 ~ 1.032 | 0.492 |
|  | Blood loss (ml/kg) | 1.006 | 0.992 ~ 1.020 | 0.434 |
|  | Surgical time (min) | 1.007 | 1.000 ~ 1.014 | 0.059 |
|  | Postoperative |  |  |  |
|  | Ki67-proliferation rate | 0.990 | 0.960 ~ 1.022 | 0.546 |
|  | WHO grade | 1.503 | 0.829 ~ 2.725 | 0.179 |
