# Supplementary material for: Effects of perioperative massive transfusion on postoperative outcomes of children undergoing brain tumor removal: a retrospective cohort study
Source: PeerJ. 2025 May 13;13:e19350. doi: 10.7717/peerj.19350 (PMC12083467; doi:10.7717/peerj.19350)
Supplement: Supplemental Information 5 [file peerj-13-19350-s005.docx]

**Table S4 Multivariable logistic regression analysis of postoperative secondary outcomes**

|  | **Variables** | **ß** | **95% CI** | ***p* Value** |
| --- | --- | --- | --- | --- |
| **Ventilation support (h)** | |  |  |  |
|  | Massive transfusion | **0.247** | 58.739 ~ 147.895 | **0.000**** |
|  | Subtentorial tumor | **0.201** | 33.063 ~ 108.173 | **0.000**** |
|  | Anaemia | 0.148 | 19.053 ~ 115.519 | 0.007** |
| **ICU stay (d)** | |  |  |  |
|  | Massive transfusion | 0.184 | 3.874 ~ 15.077 | 0.001** |
|  | Preoperative KPS score | -0.185 | -0.642 ~ -0.169 | 0.001** |
|  | Tumor number (multiple) | 0.124 | 1.070 ~ 15.784 | 0.026* |
|  | Subtentorial tumor | 0.121 | 0.563 ~ 9.902 | 0.029* |
| Hospital stay(d) |  |  |  |  |
|  | Massive transfusion | 0.118 | -0.040 ~ 9.360 | 0.053 |
|  | Preoperative KPS score | -0.117 | -0.380 ~ -0.008 | 0.042* |
|  | Subtentorial tumor | 0.245 | 4.185 ~ 11.929 | 0.000** |
|  | Crystalloids infusion (ml/kg) | 0.123 | 0.001 ~ 0.045 | 0.043* |
|  | Colloids infusion (ml/kg) | 0.142 | 0.011 ~ 0.154 | 0.024* |
|  | WHO grade | -0.154 | -3.397 ~ -0.456 | 0.011* |

KPS, Karnofsky performance scale; WHO, World Health Organization. *p<0.05 **p<0.01.
